# Antimicrobial Resistance in Humans, Animals, Water and Household Environs in Rural Andean Peru: Exploring Dissemination Pathways through the One Health Lens

**DOI:** 10.3390/ijerph18094604

**Published:** 2021-04-27

**Authors:** Stella M. Hartinger, Maria Luisa Medina-Pizzali, Gabriela Salmon-Mulanovich, Anika J. Larson, Maria Pinedo-Bardales, Hector Verastegui, Maribel Riveros, Daniel Mäusezahl

**Affiliations:** 1 School of Public Health and Administration, Universidad Peruana Cayetano Heredia, Lima 15102, Peru; maria.medina.p@upch.pe (M.L.M.-P.); hector.verastegui@swisstph.ch (H.V.); 2 Department of Epidemiology and Public Health, Swiss Tropical and Public Health Institute, 4002 Basel, Switzerland; daniel.maeusezahl@unibas.ch; 3 Swiss Tropical and Public Health Institute, University of Basel, 4001 Basel, Switzerland; 4 Institute for Nature, Earth and Energy, Pontificia Universidad Católica del Perú, Lima 15102, Peru; gsalmonm@pucp.edu.pe; 5 School of Medicine, University of Washington, Seattle, WA 98195, USA; larsona@uw.edu; 6 Instituto de Medicina Tropical Alexander von, Humboldt, Universidad Peruana Cayetano Heredia, Lima 15102, Peru; 7 School of Medicine, Universidad Peruana Cayetano Heredia, Lima 15102, Peru; maribel.riveros@upch.pe

**Keywords:** antimicrobial resistance, *E. coli*, one health, environment, child feces, Peru

## Abstract

Antimicrobial resistance (AMR) is a global public health threat, especially for low and middle-income countries (LMIC) where the threat has not been fully identified. Our study aims to describe *E. coli* AMR in rural communities to expand our knowledge on AMR bacterial contamination. Specifically, we aim to identify and describe potential dissemination routes of AMR-carrying bacteria in humans (children’s stools), community water sources (reservoirs and household sources), household environments (yard soil) and domestic animals of subsistence farmers in rural Andean areas. Our cross-sectional study was conducted in rural households in the region of Cajamarca, Peru. A total of 266 samples were collected. Thirty-four point six percent of reservoir water and 45% of household water source samples were positive for thermotolerant coliforms. Of the reservoir water samples, 92.8% were positive for *E. coli*, and 30.8% displayed resistance to at least one antibiotic, with the highest resistance to tetracycline. *E. coli* was found in 57.1% of the household water sources, 18.6% of these isolates were multidrug-resistant, and displayed the highest resistance to tetracycline (31.3%). Among samples from the children’s drinking water source, 32.5% were positive for thermotolerant coliforms, and 57.1% of them were *E. coli*. One third of *E. coli* isolates were multidrug-resistant and displayed the highest AMR to tetracycline (41.6%) and ampicillin (25%). Thermotolerant coliforms were found in all the soil samples, 43.3% of the isolates were positive for *E. coli*, 34.3% of the *E. coli* isolates displayed AMR to at least one antibiotic, and displayed the highest AMR to tetracycline (25.7%). We determined thermotolerant coliforms in 97.5% of the child feces samples; 45.3% of them were *E. coli*, 15.9% displayed multidrug resistance, and displayed the highest resistance to ampicillin (34.1%). We identified thermotolerant coliforms in 67.5% of the animal feces samples. Of those, 38.7% were *E. coli*, and 37.7% were resistant to at least one antibiotic. For all the samples, the prevalence of resistance to at least one antibiotic in the *E. coli* and *Klebsiella* spp. isolates was almost 43% and the prevalence of MDR in the same isolates was nearly 9%, yet the latter nearly doubled (15.9%) in children’s stools. Our results provide preliminary evidence for critical pathways and the interconnectedness of animal, human and environmental transmission but molecular analysis is needed to track dissemination routes properly.

## 1. Introduction

Antimicrobial resistance (AMR) has been labelled a public health threat, particularly for developing economies [1]. Treatment failures caused by AMR result in an increased risk of mortality and unnecessary burden to healthcare infrastructure, among others [2,3]. The AMR consequences for the health systems and patient health outcomes [4,5,6] are a policy challenge that directly influences community life. As the threat of AMR grows for many infectious diseases, filling the research gaps of AMR in Peru is essential.

*Escherichia coli* (*E. coli*)—and other commensal and enteric bacteria—may play a key role in the propagation of AMR genes. [7] Since fecal microbiota serves as the reservoir of these genes [8], which could be transferred to pathogenic organisms [9], the risk for resistant infections in the community increases. Multiple studies in Peru reported growing rates of AMR in commensal and enteric bacteria [10,11,12]. Many AMR genes significant in clinical settings are believed to have originated from non-pathogenic bacteria [13]. Resistance to ampicillin, cotrimoxazole, tetracycline, chloramphenicol, nalidixic acid and ciprofloxacin was reported in *E. coli* recovered among children in a periurban population in Peru [14]. Likewise, there is evidence of AMR in enterobacteria found in children across different environments: rural towns of the Amazon and Andean regions, periurban slums in desert coastal cities, and villages in the Amazon region [15,16,17]. Nevertheless, most of the evidence for AMR in human populations is focused on urban and periurban settings rather than rural areas [15]. On the other hand, extended-spectrum β-lactamases (ESBL) production is common in *E. coli* and other enterobacteria. ESBL-producing microorganisms cause high-mortality infections, given that ESBL hydrolyses the therapeutically important carbapenems and other beta-lactam antibiotics. As a result, therapeutic options available are greatly narrowed down [18].

The increase in AMR worldwide is a result of inappropriate antibiotics prescription by healthcare providers, treatment adherence among patients who do not use the antibiotics as prescribed, over-the-counter availability of antibiotics without a prescription [19], the inadequate use of antimicrobials in animal production, and the absence of integrated surveillance programs for antimicrobial resistance [20], which should focus on humans, animals and the environment [21]. In addition, unhygienic living conditions and the exposure to untreated or poorly treated water aggravate the AMR problem in developing countries [18], especially in rural settings [17].

The amount of antimicrobials used in animal production exert environmental pressure favoring the generation, and spread of AMR bacteria through different routes, mainly soil, water, food and farm animals [19,22,23]. Food-producing animals are commonly carriers of AMR and MDR bacteria, causing dissemination of AMR into humans—farm workers being at a higher risk—and ecosystems [7]. Sewage and surface water contaminated with sewage effluents are commonly used in the irrigation of crops, and animal drinking supply, driving the spread and maintenance of AMR bacteria in the environment [24]. Other factors prompting AMR include environmental contamination with industrial effluents containing metals and biocides, and the use of pesticides in agriculture, which can select for AMR genes in bacteria [25]. The lack of water treatment in the households, [17] consumption of conventional chicken—raised with antibiotics—[16] and the presence of antibiotics in dairy products [26] are factors favoring the dissemination of AMR in Peruvian ecosystems.

The One Health concept recognizes that “human health and animal health are interdependent and bound to the health of the ecosystems in which they exist” [27]. It has been specifically proposed as a framework to address AMR by the World Health Organization (WHO), the Food and Agriculture Organization, and the World Organization for Animal Health [28,29]. The WHO 2018 report on antimicrobial use and AMR, recommended their surveillance under a One Health approach [30]. While there are existing regulations on the use of antimicrobials for animal production in Peru, these are not closely enforced [26]. Regulations center on the types of antibiotics used and the detection of residue in products for human consumption and are also included in the National Plan to Confront Antimicrobial Resistance [31]. Furthermore, there are several additional barriers to implementing this concept, including parallel surveillance systems on human and animal health and ignoring the potential role of wildlife species [32,33].

Adopting a One Health approach [34], this study aimed to describe AMR in San Marcos, Cajamarca, building on our current knowledge on AMR bacterial contamination [17]. We aimed to investigate the presence of AMR thermotolerant coliforms (i.e., *E. coli, Klebsiella, Enterobacter, Citrobacter*) in humans (stool samples from children), environments like community water sources (reservoirs and household sources), household environments (yard’s soil), and domestic animals of subsistence farmers (e.g., pigs, poultry); and to propose carriage and dissemination routes of AMR bacteria in the household environment. Our study’s findings could be useful for policy makers on this critical issue in the context of rural Peru and may also be applicable to other rural areas in the Andean region.

## 2. Methods

### 2.1. Study Site

Our study was conducted in rural homes of the San Marcos and Cajabamba provinces in the region of Cajamarca, Peru. This region is located approximately 2200–4000 m above sea level. Households typically obtain drinking water from central community reservoirs that are piped directly into individual homes or their courtyards. Among homes in this community, the preferred method of household water treatment is boiling [35], and most homes own livestock. Most small animals such as pigs and birds roam freely around household environs.

### 2.2. Study Design

Using a cross-sectional design, we purposely selected households with high AMR levels in the child’s drinking water. These households came from among 102 communities in the northern highlands that had previously participated in a community-randomized controlled trial [36]. Homes that had a child under five years, a drinking water sample positive for *E. coli* with AMR, and homes keeping farm animals (mainly pigs and fowl) (unpublished data) were targeted and invited to participate in this study. All households were enrolled between May and June 2019.

#### 2.2.1. Sample Collection

Trained fieldworkers visited each participating household (N = 40) in the morning on two consecutive days to collect stool samples from children and animals, drinking water samples, and soil samples from the household’s yard. We also collected water samples from a community water source. In addition, a household questionnaire was used to identify potential risk and protective factors to AMR, and corroborate AMR dissemination pathways in rural settings. A total of 266 samples were collected. For further pathogen identification, all samples were stored for up to three days in peptone media vials and were transported for analysis to the Enteric Diseases and Nutrition and Antibiotic Resistance Laboratory at the Tropical Medicine Institute, Universidad Peruana Cayetano Heredia, Lima.

#### Human and Animal Fecal Samples

Animal sample collection: We collected two rectal or cloacal swabs of fresh stool samples, ideally one from a domestic animal (dog, cat) and one from a farm animal (cow, pig, fowl). If the combination was not possible, collecting the same animal type was permitted. One veterinarian and one field worker were responsible for collecting the samples. The handling of the animal was done by the owner (to avoid additional stress on the animal) and a trained fieldworker while the veterinarian was responsible for swabbing the animal for the sample. All animals were handled with care. We transported the samples using a cooled envelope to the field laboratory within 4 h of collection. The specimens were stored in Cary Blair transport media and refrigerated at −4 °C. The samples were sent weekly to Lima for laboratory analysis.

#### Environmental Samples

Water samples: We collected two water samples, one obtained from the child’s main drinking water source—which could have been stored and/or home treated—and the second from the household’s primary water source (i.e., the tap or outside standpipe). If the household only had one of the two potential types of water sources at the time of the visit, the available water source was collected twice. Water samples were also collected from the community water reservoir. The reservoir could supply more than one community.

All samples were transported back to the field station within 8 h of collection, and analysed using the membrane-filtration method of Oxfam DelAgua Water Testing Kit, product code 14867 [37].

Soil samples: We collected five shallow (less than 5 cm depth) soil samples of different random points (5 g of soil per sample) from the main playing area of the child (or from the area where the child spent the most time), using sterile metallic spoons. The samples were placed in labelled Ziplock sterile bags and transported back to the field station.

#### 2.2.2. Laboratory Analysis of Samples

Human and animal samples: *Enterobacteriaceae* isolates were identified using CHROMagar Orientation (CHROMagar, France) and conventional microbiological methods according to Biochemical Tests for Identification of Medical Bacteria [38].

Water samples (reservoir and drinking water samples) were analysed for thermotolerant (faecal) coliforms using the membrane-filtration method of the Oxfam DelAgua Water Testing Kit. We incubated the samples at 44 °C ± 0.5 °C, from 14 to 16 h in lauryl sulphate broth. Samples were evaluated according to the kit’s instructions, counting the yellow colonies forming units (CFU) in the first 15 min as indicative of thermotolerant bacterial growth. We stored colonies with similar morphology in peptone media vials and sent the vials weekly to Lima for the antibiotic susceptibility testing and ESBL detection and molecular confirmation.

Soil samples were homogenised in the San Marcos field station, and 1 g of each sample was transferred to Luria Bertani Broth (25 mL). The samples were incubated at 37 °C for 24 h, stored at 4 °C and were sent weekly to Lima for the antibiotic susceptibility testing and ESBL detection and molecular confirmation.

#### Antibiotic Susceptibility Testing

The antibiotic resistance pattern was determined against fourteen antibiotics using the Kirby–Bauer disk diffusion method following the Clinical and Laboratory Standards Institute (CLSI) guidelines [39]: nalidixic acid (30-µg disk), ciprofloxacin (5-µg disk), chloramphenicol (30-µg disk), gentamicin (10-µg disk), tetracycline (30-µg disk), trimethoprim-sulfamethoxazole (25-µg disk), amoxicillin-clavulanic acid (30-µg disk), ampicillin (10-µg disk), cefotaxime (30-µg disk), cefepime (30-µg disk), aztreonam (30-µg disk), cefoxitin (30-µg disk), ceftriaxone (30-µg disk), and imipenem(10-µg disk). Antibiotic susceptibility testing was performed for all isolated bacteria.

#### Extended Spectrum Beta Lactamases (ESBL) Detection and Confirmation

Phenotypic detection of ESBL bacteria: Antibiotic susceptibilities for all bacterial isolates were tested using the Jarlier method [40] for the following antibiotics: aztreonam (5-µg disk), ceftazidime (30-µg disk), cefotaxime (30-µg disk), ceftriaxone (30-µg disk), amoxicillin-clavulanic acid (30-µg disk) and cefepime (30-µg disk), and confirmed by combined disks.

Molecular confirmation of ESBL genes: *E. coli* isolates displaying phenotypic ESBL activity were tested by conventional polymerase chain reaction (PCR) to identify the genes *bla_TEM_, bla_SHV_*, and *bla_CTX-M_* [41,42,43,44]. Within the *bla_CTX-M_* group, the following subgroups were determined: *bla_CTX-M-2_, bla_CTX-M-3_, bla_CTX-M-8_, bla_CTX-M-9_*, and *bla_CTX-M-10_*. The primers used are shown in Supplementary Table S1. Identified ESBL genes were not sequenced for allelic variants.

#### 2.2.3. Questionnaires

We created and applied a questionnaire that considered the One Health approach to identify the transmission pathways to explain AMR dissemination. Trained fieldworkers applied the questionnaire to collect information on AMR dissemination pathways, household hygiene practices, household water management, recent antibiotic use by household members, animal management, and agricultural practices to identify routes for the spread of AMR in rural settings.

### 2.3. Data Analysis

The data was entered in the Census and Survey Processing System (CS Pro 6.3) and exported to Stata 15 Statistical software (STATA CORP, College Station, TX, USA) for analysis. We carried out a descriptive analysis, and compared the frequencies of AMR bacterial types between human, animal, and environmental sources. We assessed AMR patterns identified in the household drinking water samples and animal samples from the same site and water sources from the area.

### 2.4. Ethics

Human (418-16-18) and Animal (010-03-20) ethical review boards from the Universidad Peruana Cayetano Heredia approved the study. Each participant signed a written informed consent, agreeing to participate in our study.

## 3. Results

### 3.1. AMR Dissemination Pathways in Rural Settings

**Figure 1:**
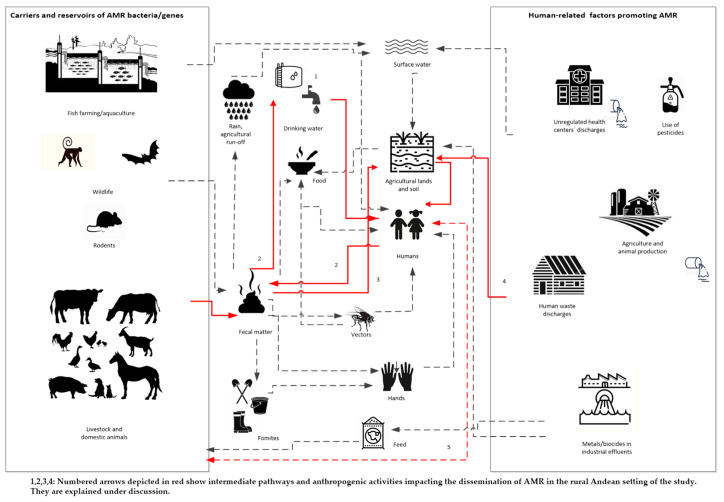
Identified dissemination pathways in Peruvian rural settings applying a One Health lens.

Using the One Health approach, we tried to establish a AMR bacteria dissemination pathway, and evaluated how the AMR bacteria could spread, and how AMR drivers would prompt the dissemination in Cajamarca’s rural setting (Figure 1). We found evidence for specific pathways, and these are represented in red solid lines in Figure 1.

### 3.2. Setting Description

The main demographics, household’s characteristics, household’s water treatment, and animal management and treatment, are found in Table 1. 72.5% of homes had access to a piped water system and 20% to the yard or household premises. Both systems are a gravity-based piped water supply system. For drinking water, 27.5% of participants consumed water directly from the faucet without any treatment, 60% declared boiling the water and a small proportion (12.5%) reported treating the water with chlorine or bleach. For animal handling and treatment, 72.5% of the households responded that they received antibiotics as part of their last treatment. The main antibiotic brands used were “Ciclosona” (50%) and “Biomizona” (21.8%), both containing oxytetracycline and an anti-inflammatory drug. More than 80% of the homes reported that they got the antibiotics from a veterinary doctor, technician, or a local veterinary store.

### 3.3. Water Samples

In total, we collected 106 water samples, 26 from the reservoir, 40 from the main’s household water source, and 40 from the child’s drinking water source. As shown in Table 2, nine out of the 26 water reservoir samples (34.6%) were positive for thermotolerant coliforms. From these positive samples, we obtained a total of 14 bacteria isolates, and 92.8% were positive for *E. coli*. For the main household water samples (collected from faucet or pitcher), 18 out of 40 (45%) were positive for thermotolerant coliforms. We obtained a total of 28 thermotolerant bacterial isolates, and 82.1% of them were *Enterobacteriaceae*. Of the enterobacteria isolates, 57.1% were *E. coli*, 10.7% *Klebsiella* spp. and 14.8% were *Enterobacter* spp. Thirteen out of the 40 (32.5%) child’s drinking water samples were positive for thermotolerant coliforms, and a total of 27 thermotolerant bacteria were isolated from these positive samples. *Enterobacteriaceae* represented 74% of all the isolates, and 44.4% of the enterobacteria isolates were *E. coli*, 14.8% were *Klebsiella* spp. and 14.2% *Enterobacter* spp. (Table 2).

We determined the phenotypic antibiotic resistance profile for *Escherichia coli* and *Klebsiella* spp. isolates. From the 13 *E. coli* isolates found in the reservoir’s water, 30.8% displayed resistance to at least one antibiotic. The *E. coli* antibiotic profile showed the highest resistance to tetracycline. All *Klebsiella* spp. isolates from the main household’s water and child’s drinking water displayed resistance to at least one antibiotic, showing the highest resistance to ampicillin. Multidrug resistance was displayed in 33.3% and 25% of the *Klebsiela* spp. isolates for the main household’s water and child’s drinking water, respectively (Table 3). *E. coli* also showed the highest resistance towards tetracycline (31.3%), ampicillin and nalidixic acid (18.8%) in the main household’s water source. We found the highest resistance to tetracycline (41.6%) and ampicillin (25%) in the child’s drinking water source (Table 3).

### 3.4. Soil Samples

All soil was positive for thermotolerant coliforms. We obtained 83 isolates from the samples. Of these, 43.3% were identified as *E. coli*, 4.8% *Klebsiella* spp., 24.1% *Enterobacter* spp. and 9.6% *Citrobacter* spp. (Table 4).

Some 36 *E. coli* isolates were found in the soil samples of which 33.3% displayed resistance to at least one antibiotic and one showed multidrug resistance. From the *Klebsiella* spp. isolates, 75% displayed resistance to at least one antibiotic, but no multidrug resistance was observed. The *E. coli* antibiotic profile displayed highest resistance to tetracycline (25%) and ampicillin (11.1%), and *Klebsiella* spp. showed the highest resistance to ampicillin (Table 5).

### 3.5. Child Fecal Samples

97.5% of the child fecal samples were positive for thermotolerant coliforms (Table 4). We obtained a total of 98 thermotolerant bacteria isolates. Of these, almost half of the samples had *E. coli* (45.3%), followed by *Klebsiella* spp. (11.3%)*, Citrobacter* spp. (9.2%) and *Enterobacter* spp. (6.2%).

We carried out antibiotic resistance profiling for *Escherichia coli* and *Klebsiella* spp. isolates in the child fecal samples (Table 5). From all *E. coli* isolates, 52.3% displayed resistance to at least one antibiotic and 15.9% were multidrug-resistant; 54.6% of the *Klebsiella* spp. isolates displayed resistance to at least one antibiotic, but we did not find multidrug resistance. The highest resistance for the *E. coli* isolates was to ampicillin (34.1%) and tetracycline (25.0%) and the highest resistance for *Klebsiella* spp. was to ampicillin (54.5%).

### 3.6. Animal Fecal Samples

Of the 80 animal fecal samples, 67.5% were positive for thermotolerant coliforms. We obtained a total of 116 thermotolerant bacteria isolates, and they were identified as *E. coli* (38.7%), *Klebsiella* spp. (5.1%), *Citrobacter* spp. (16.3%) and *Enterobacter* spp. (4.3%) (Table 4).

We performed antibiotic resistance profiling for *Escherichia coli*, Klebsiella spp. *Citrobacter* spp. and *Enterobacter* spp. in the isolates. From the isolates 37.7% of *E. coli*, 50% of *Klebsiella* spp. and 60% of the *Enterobacter* spp. isolates displayed resistance to at least one antibiotic. None were multidrug-resistant (Table 5).

### 3.7. Multidrug Resistance Profiles

Among the *E. coli* isolates obtained from the child’s feces, child’s drinking water source, household’s main water source and soil, 13.9% (15/108) were resistant to three or more classes of antibiotics [46]. Most of them were resistant to ampicillin, trimethoprim-sulfamethoxazole, tetracycline, nalidixic acid, and ciprofloxacin. Only one isolate of *E. coli* was identified as a carrier of ESBL (Table 6).

### 3.8. Detection of ESBL Resistance Genes

We identified two bacterial isolates harbouring ESBL genes. One was an *E. coli* isolate from a water sample, and one was a *Shigella* spp. isolate from a dog faecal sample. The ESBL *E. coli* isolate carried the *bla_TEM_, bla_CTX-M-U_*, and *bla_CTX-M-8_* genes; and the ESBL *Shigella* spp. isolate carried the *bla_TEM_, bla_CTX-M-U_*, and *bla_CTX-M-3_* genes. PCR amplification of β-lactamase genes for both samples are found in Supplementary Figure S1.

## 4. Discussion

Our study is among the first to investigate specific aspects related to AMR’s spread in the Andean region in Peru. Adopting the One Health lens provided a unique and important insight into the complex, interlinked problem between human, animal, and environment health [47].

Our results provide descriptive evidence for the pathways shown in red in Figure 1. AMR thermotolerant bacteria—mainly *E. coli*—were found in children’s stools and animal faeces, and they were also detected in the reservoir water, the household’s and child’s drinking water sources; as well as in the soil from the household’s yard. For all the samples, the prevalence of resistance to at least one antibiotic in the *E. coli* and *Klebsiella* spp. isolates was almost 43% and the prevalence of MDR in the same isolates was nearly 9%, yet the latter nearly doubled (15.9%) in children’s stools.

Our finding of thermotolerant coliforms in the reservoir’s water indicates recent fecal contamination [48]. 34.6% of reservoir water samples were positive for thermotolerant coliforms, with counts above the Peruvian and WHO threshold guidelines (0 CFU in 100 mL) [48,49]. We provide two likely explanations for these findings. Poor reservoir infrastructure and/or the distribution network results in contamination, possibly with animal faeces. In Peruvian rural Andean settings, about 30% of water storage and supply systems are older than 20 years, and some 20% have collapsed [50]. Another potential explanation is that agricultural run-off, rain, surface or underground water containing animal or human fecal matter seep into the system [48,51]. Further, inadequate water supply management, infrequent cleaning or disinfection, irregular treatment (automated chlorination systems or manual chlorination) of the reservoir, and/or the lack of a maintenance backlog and the use of old materials are also frequent concerns [50,52]. According to the Peruvian Ministry of Housing, Construction and Sanitation [50], only 6.9% of water storage and supply facilities apply proper treatment guaranteeing water safety in rural Peru. In all Cajamarca, including the San Marcos Province, reservoirs do not have an automated disinfection system; most use manual chlorination and are managed unreliably by the community water supply and irrigation committees (JASS) [52]. In fact, an earlier study in the same area found that the spring water stored at the reservoir was unfiltered, untreated, and chlorination was performed infrequently [53]. Given that 27.3% of all *E. coli* isolates from the reservoirs’ water displayed AMR and had faecal origin, the water distribution network could play an important role in spreading AMR in the population (See Figure 1, pathway 1).

In the households, we found that 25% of the households’ heads reported consuming water directly from the faucet or bucket without any previous household water treatment (HWT), exposing residents to potential contamination in case of failures in the central water treatment facility. Most households reported boiling or adding chlorine as their preferred HWT methods; however, it is most likely that the real proportion of homes treating their water regularly is much lower, based on the findings of this study and previous ones from the area [35]. We found that nearly half of the household water samples were positive for thermotolerant coliforms, and of the 57.1% *E. coli* isolates, 18.6% showed multidrug resistance. It is not clear whether the home-treated water is being recontaminated from bacteria found within the household environs or the recontamination is caused by inadequate storage. However, it could also be due to poor hygienic practices in the household, lack of handwashing, and free-roaming animals and vectors. Thus, the AMR bacterial isolates in drinking water could originally come from human or animal waste [54], as shown in pathway 2, Figure 1.

We found that the AMR profiles show a relationship with the most commonly used antibiotics in the area. Oxytetracycline was the most common antibiotic used for animal treatment reported by the household head. Coincidently, the highest resistance for the *E. coli* isolates in animals’ faeces was tetracyclines, and similar resistance profiles were observed in all the water samples (reservoir and drinking water samples). This underscores the hypothesis that faeces are contaminating water within the water delivery system. Tetracyclines are a family of antibiotics widely used in veterinary medicine and animal production; compared to other antibiotics used in livestock farming, they are applied in greater amounts and tend to persist in the environment for longer periods [55]. Tetracycline use and resistance have been reported in other rural environments with animal production activity [26,56,57]. Children’s drinking water samples also displayed resistance to ampicillin, which is the most common antibiotic used in the area for treating childhood illnesses. This indicates that treated drinking water for children’s consumption could be recontaminated with children’s feces due to mismanagement and poor personal hygiene within the home (Figure 1, pathway 2).

Multidrug-resistant and thermotolerant coliform bacteria were prevalent in the study area. We found that one third of all *E. coli* isolates from the child’s drinking water were positive for MDR. According to the WHO list of critically important antimicrobials classification, third and fourth generation cephalosporins, quinolones and tetracycline in the child’s drinking water could indicate a severe public health risk for children in rural areas, given the lack of treatment options for multidrug-resistant infections. Multidrug resistance in coliforms is escalating worldwide, and it may be explained by their high tendency to transfer and receive AMR genes horizontally [58]. In a recent study in the rural Andean regions of Peru, Larson et al. [17] found a lower percentage (19.7%) but still alarming frequency of multidrug resistant *E. coli* in children’s drinking water just four years ago. It is unclear whether the propagation of resistant bacteria and/or the spread of AMR genes are rising in this rural area. The higher percentage of MDR bacteria found among *E. coli* isolates and bacteria carrying ESBL genes (*bla_TEM_*, *bla_CTX-M-U_* and *bla_CTX-M-8_*) in children’s drinking water compared to the main household water, could be due to poor water treatment and hygiene practices, inappropriate use (unpublished data) or contaminated storage containers [59]. Nearly 59% of the households that reported treating their water, also reported storing it in different types of containers; the use of wide-mouth containers increased the possibility of recontamination (unpublished data). Similar findings are described in a study investigating drinking water samples in rural households in Ecuador [60].

The high prevalence of thermotolerant coliforms found in the soil indicates significant fecal contamination, given that most animals roam freely in the courtyard and in the community. Evidence shows that in rural areas, soil fecal contamination is mainly attributed to animals [61,62]. The environs of family households and farms may be more affected by AMR due to the presence of animal manure. In many cases, animal manure is used to fertilize crops, increasing the chances of AMR spread to farmland and produce [56]. The prevalence of resistance to any antibiotic in *E. coli* and *Klebsiella* spp. in animal faeces was 37.7% and 50%, whereas in soil it was 33.3% and 75%, respectively, supporting pathway 3 in Figure 1. The finding of ESBL genes (*bla_TEM_*, and *bla_CTX-M-3_*) on an *Shigella* spp. isolate from a dog illustrates the importance of strengthening surveillance programmes for MDR to gain a better understanding of community source dissemination. Given that humans are *Shigella* spp. main reservoir [63], its finding in a dog flags the possibility of transmission from humans to animals (pathway 5, Figure 1). We found evidence in South America of the presence of *E. coli* carrying ESBL genes in dog feces in public parks [64]. Another possible source of soil contamination is water run-offs from poorly designed and poorly maintained pit latrines. Fifty-three percent of the households in the study area own and use pit latrines [36]. Pit latrines seep nightsoil into the ground and potentially contribute to the propagation of AMR bacteria in the environment [65]. Pathway 4 in Figure 1 seems plausible, given that in children’s stools the prevalence of resistance to any antibiotic in *E. coli* and *Klebsiella* spp. was 52.3% and 54.6%, respectively. The finding of multidrug resistance in 15.9% of all *E. coli* isolates from the children’s faeces indicates a high public health risk and calls for AMR surveillance to control the exposure to AMR bacteria in rural Andean settings like ours. However, no ESBL genes were found in these samples.

## 5. Limitations

By intentionally focusing on studying AMR high-level households, we biased our estimates to be higher than what could potentially be expected in the average community.

Nevertheless, this decision allowed us to establish the principal pathways of transmission. We must assume that in less contaminated communities, those routes pertain as well and contribute to the AMR problem, but, due to their low numbers, they are difficult to detect.

## 6. Conclusions

The AMR problem in Peru is still largely underexplored, especially in rural regions. Using a One Health perspective to identify transmission pathways for AMR and acknowledging the convergence of animal, human, and environment health dimensions in the spread, we identified critical pathways of infection for rural settings. Our epidemiological findings demonstrate the interconnectedness of animal, human and environmental transmission. However, molecular analysis is needed to elucidate if the isolates found in each type of sample are clones, proving that the same AMR bacteria strains are shared. The high prevalence of AMR and MDR bacteria in children, soil, and water samples is alarming. Specifically for animal and child feces, we found that the resistance profiles seem to relate to the antibiotics most commonly used for treatment. This poses a critical public health threat as it can limit the use of these first line drugs in future. Drinking water is a neglected potential source of community exposure to antibiotic-resistant organisms. The presence of ESBL genes in drinking water and animal faeces samples show the anthropogenic origins of AMR. A standard microbiological water quality testing and management is needed and where protocols for the management and specific treatment of delivery networks exist, they need to be reinforced to reduce the current risk exposure to these harmful pathogens.

## Figures and Tables

**Table 1:** Descriptive statistics of households’ demographic characteristics, water supply and treatment, animal keeping.

	N	Mean [SD] or % (N)
Demographic characteristics	40	
Number of inhabitants per household		5.0 [1.44]
Number of children under 6 per household		1.4 [0.53]
Household characteristics		
Adobe wall type		
- Coated adobe or rammed earth		60 (24)
- Uncoated adobe or rammed earth		22.5 (9)
Latrines w/o ventilation		
- Septic tank		22.5 (9)
- Latrine		75 (30)
Piped water supply		
- Public water supply system/piped water in the house		72.5 (29)
- Public water supply system/piped water outside the house		20 (8)
- Public water supply system/piped water outside the house but inside the building		2.5 (1)
- Surface water, spring		5 (2)
Energy source		
- Electricity		87.5 (35)
- Candle		5 (2)
- Solar Panel		7.5 (3)
Household Water Treatment		
Boiling		60 (24)
Chlorine or bleach		12.5 (5)
None		27.5 (11)
Animal Management and treatment		
The last time the animal was treated; did the animal receive any antibiotic?		72.5 (29)
Antibiotic used for the treatment		
- Amoxicillin		3.1 (1)
- “Biomizona“ ^1^		21.8 (7)
- “Ciclosona” ^2^		50 (16)
- “Emicina” ^3^		3.1 (1)
- “Hipradoxi S” ^4^		3.1 (1)
- “Hipralona” ^5^		6.2 (2)
- “Quinolaba” ^5^		6.2 (2)
- “Tylogen” ^6^		6.2 (2)
Where did they get the antibiotic?		
- Directly form a veterinarian		18.7 (6)
- Directly from a veterinarian technician		50 (16)
- From a neighbour or relative		3.1 (1)
- At a local veterinary store		18.7 (6)
- At a veterinary store in the area		0
- At a pharmacy		0
- Other place		9.3 (3)

^1^ Brand name for a commercial formulation of oxytetracycline and benzydamine, ^2^ brand name for a commercial formulation of oxytetracycline and dexamethasone, ^3^ brand name for oxytetracycline, ^4^ brand name for doxycycline, ^5^ brand names for enrofloxacin, ^6^ brand name for a commercial formulation of Gentamicin and Tylosin.

**Table 2:** Descriptive statistics of bacterial contamination, frequency and type of thermotolerant coliform identified from all water sources.

Coliforms (Count)	Water from Reservoir (N = 26) % (N)	Main Household’s Water (N = 40) % (N)	Child’s Drinking Water (N = 40) % (N)
Thermotolerant coliform count (IQR 1st–3rd Quantile)	0–3.75	0–10.5	0–9.5
Thermotolerant coliform (CFU/mL)—mean (SD)	14.3 (59.2)	36.2 (108.4)	104.1 (373.5)
Total positive thermotolerant sample	34.6 (9)	45 (18)	32.5 (13)
Total thermotolerant bacterial isolates *	N = 14 % (n)	N = 28 % (n)	N = 27 % (n)
Total positive *Enterobacteriaceae* isolates	92.8 (13)	82.1 (23)	74.0 (20)
*E. coli*	92.8 (13)	57.1 (16)	44.4 (12)
*Klebsiella* spp.	0	10.7 (3)	14.8 (4)
*Enterobacter* spp.	0	14.8 (4)	14.2 (4)

* Correspond only to the positive enterobacteria isolates.

**Table 3:** *Escherichia coli* and *Klebsiella* spp. antibiotic resistance profile to a panel of antibiotics, water type (reservoir, main household water source, child drinking water), and proportion of multidrug-resistant isolates.

	Water from Reservoir	Main Household’s Water		Child’s Drinking Water	
	*E. coli* N = 13	*E. coli* N = 16	*Klebsiella* spp. N = 3	*E. coli* N = 12	*Klebsiella* spp. N = 4
Antibiotic	Resistance % (N)	Resistance % (N)		Resistance % (N)	
Amoxicillin-clavulanic acid	0	0	0	0	0
Ampicillin	0	18.8 (3)	100 (3)	25 (3)	100 (4)
Aztreonam	0	0	0	0	0
Cefotaxime	15.4 (2)	0	0	8.3 (1)	0
Cefoxitin	0	0	0	0	0
Chloramphenicol	7.7 (1)	12.5 (2)	0	25 (3)	0
Ciprofloxacin	0	6.3 (1)	33.3 (1)	16.7 (2)	25 (1)
Gentamicin	0	6.3 (1)	0	8.3 (1)	0
Nalidixic acid	0	18.8 (3)	0	16.7 (2)	0
Trimethoprim-sulfamethoxazole	0	0	33.3 (1)	8.3 (1)	25 (1)
Tetracycline	15.4 (2)	31.3 (5)	33.3 (1)	41.7 (5)	25 (1)
Ceftriazone	0	0	0	11.1 (1)	0
Cefepime	0	0	0	8.3 (1)	0
Imipenem	0	0	0	0	0
AMR to at least one antibiotic ^1^	30.8(4)	43.8 (3)	100 (3)	41.7 (5)	100 (4)
Multidrug resistance ^2^	0 (0)	18.6 (3)	33.3 (1)	33.3 (4)	25 (1)

^1^ Antimicrobial resistance (AMR) is defined as “the ability of a microorganism to stop an antimicrobial from working against it. As a result, standard treatments become ineffective; infections persist and may spread to others” [45]. ^2^ Multidrug resistance is defined as resistance to three or more classes of antibiotics [46].

**Table 4:** Bacterial contamination by frequency and type of thermotolerant coliform in household and agricultural soil and animal and human feces.

Coliforms	Soil (N = 40) % (n)	Child Faeces (N = 40) % (n)	Animal Faeces (N = 80) % (n)
Thermotolerant coliforms	100 (40)	97.5 (39)	67.5 (54)
Total thermotolerant bacterial isolates	N = 83 % (n)	N = 98 % (n)	N = 116 % (n)
*E. coli*	43.3 (36)	45.3 (44)	38.7 (45)
*Klebsiella* spp.	4.8 (4)	11.3 (11)	5.1 (6)
*Enterobacter* spp.	24.1 (20)	6.2 (6)	4.3 (5)
*Citrobacter* spp.	9.6 (8)	9.2 (9)	16.3 (19)

**Table 5:** *Escherichia coli* and *Klebsiella* spp. antibiotic resistance profile to a panel of antibiotics, per sample type (soil, child and animal feces), and proportion of multidrug-resistant isolates.

	Soil		Child Faeces		Animal *Faeces*	
	*E. coli* N = 36	*Klebsiella* spp. N = 4	*E. coli* N = 44	*Klebsiella* spp. N = 11	*E. coli* N = 45	*Klebsiella* spp. N = 6
Antibiotic	Resistance % (N)		Resistance % (N)		Resistance % (N)	
Amoxicillin-clavulanic acid	0	25 (1)	0	9.1 (1)	4.4 (2)	0
Ampicillin	11.1 (4)	75 (3)	34.1 (15)	54.5 (6)	11.1 (5)	50 (3)
Aztreonam	0	0	2.3 (1)	0	2.2 (1)	0
Cefotaxime	0	0	0	0	0	0
Cefoxitin	2.8 (1)	25 (1)	0	9.1 (1)	4.4 (2)	0
Chloramphenicol	2.8 (1)	0	4.5 (2)	0	11.1 (5)	0
Ciprofloxacin	5.5 (2)	0	11.4 (5)	0	8.8 (4)	0
Gentamicin	0	0	2.3 (1)	0	0	0
Nalidixic acid	5.5 (2)	0	13.6 (6)	0	20 (9)	0
Trimethoprim-sulfamethoxazole	5.5 (2)	0	20.5 (9)	9.1 (1)	11.1 (5)	0
Tetracycline	25.0 (9)	0	25.0 (11)	9.1 (1)	26.6 (12)	0
Ceftriazone	0	0	2.3 (1)	0	0	0
Cefepime	0	0	0	0	0	0
Imipenem	0	0	0	0	0	0
AMR^1^	33.3 (12)	75 (3)	52.3 (23)	54.6 (6)	37.7 (17)	50 (3)
MDR^2^	2.8 (1)	0 (0)	15.9 (7)	0 (0)	0 (0)	0 (0)

**Table 6:** Profile of all multidrug-resistant *E. coli* isolates from different sources.

Source Type ***	MDR	Antimicrobial Class **
Child Water	AMP, TE y C	Penicillin
Child Water	SXT, TE, C	Sulfonamides, tetracycline, quinolone
Child Water	NA, CIP y TE	Quinolone, tetracycline
Child Water *	AMP, CTX, CRO, FEP, NA, CIP, TE, C y CN	Penicillin, 3rd & 4th generation cephalosporin, quinolone, tetracycline
HH Water Source	NA, TE, C y CN	Quinolone, tetracycline
HH Water Source	NA, CIP, SXT, TE, C	Quinolone, sulfonamides, tetracycline
HH Water Source	AMP, SXT, TE	Penicillin, quinolone, tetracycline
Soil	AMP, NA, CIP, SXT, TE, C	Penicillin, quinolone, sulfonamides, tetracycline
Child faeces	AMP, NA, CIP, SXT, TE y CN	Penicillin, quinolone, sulfonamides, tetracycline
Child faeces	AMP, SXT, TE	Penicillin, sulfonamides, tetracycline
Child faeces	AMP, TE, C	Penicillin, tetracycline, quinolone
Child faeces	AMP, SXT, TE	Penicillin, sulfonamides, tetracycline
Child faeces	CRO, CIP, SXT	3rd generation cephalosporin, quinolone, sulfonamides
Child faeces	AMP, SXT, TE	Penicillin, sulfonamides
Child faeces	AMP, NA, CIP, SXT, TE	Penicillin, quinolone, sulfonamides, tetracycline

AMP: ampicillin, STX: trimethoprim-sulfamethoxazole, NA: nalidixic acid, TE: tetracycline, CIP: ciprofloxacin, C: chloramphenicol, CN: gentamicin, CTX: cefotaxime, FEP: cefepime and CRO: ceftriaxone. * *E. coli* isolates harboring ESBL, ** as per WHO antimicrobial class classification. *** HH: household.

## Data Availability

The data presented in this study are available on request from the corresponding author.

## Supplementary Materials

The following supplementary files are available online at: https://www.mdpi.com/article/10.3390/ijerph18094604/s1. Table S1: Primers used for the detection of genes encoding the production of ESBL. Figure S1: PCR amplification of β-lactamase genes harboring blaTEM, blaCTX-M-8 and blaCTX-M-3 genes in *Shigella* spp. (S1) isolate from a dog faecal sample and *E. coli* (S2) isolate from a water sample. L, 100 bp DNA ladder, C-, negative control, C+, positive control. (A) blaTEM (1150 bp), (B) blaCTX-M-3 (1017 bp), (C) blaCTX-M-8 (800 bp) amplification products.

## Abbreviations

AMR	Antimicrobial resistance
LMIC	Low- and middle-income countries
CFU	colonies forming units
CLSI	Clinical and Laboratory Standards Institute
JASS	community water supply and irrigation committee
HWT	Household water treatment
MDR	Multidrug-resistant
