# Supplementary material for: Antimicrobial Resistance in Humans, Animals, Water and Household Environs in Rural Andean Peru: Exploring Dissemination Pathways through the One Health Lens
Source: Int J Environ Res Public Health. 2021 Apr 27;18(9):4604. doi: 10.3390/ijerph18094604 (PMC8123641; doi:10.3390/ijerph18094604)
Supplement: Supplementary file 1 [file ijerph-18-04604-s001.zip › ijerph-1118792-supplementary.pdf]

Table S1: Primers used for the detection of genes encoding the production of ESBL

| **Gene** | | **Sequence (5'→ 3')** | **Size (bp)** | **Reference** |
|---|---|---|---|---|
| $bla_{TEM}$ | F | ATTCTTGAAGACGAAAGGGC | 1150 | [40] |
| | R | ACGCTCAGTGGAACGAAAAC | | |
| *bla*$_{SHV}$ | F | CACTCAAGGATGTATTGTG | 885 | [41] |
| | R | TTAGCGTTGCCAGTGCTCG | | |
| *bla*$_{OXA}$ | F | ACACAATACATATCAACTTCGC | 813 | [42] |
| | R | AGTGTGTTTAGAATGGTGATC | | |
| *bla*$_{CTX-M-U}$ | F | CGATGTGCAGTACCAGTAA | 585 | [43] |
| | R | TTAGTGACCAGAATCAGCGG | | |
| *bla*$_{CTX-M-2}$ | F | ATGATGACTCAGAGCATTCG | 876 | [43] |
| | R | TCAGAAACCGTGGGTTAC | | |
| *bla*$_{CTX-M-3}$ | F | GTTACAATGTGTGAGAAGCAG | 1017 | [43] |
| | R | CCGTTTCCGCTATTACAAAC | | |
| *bla*$_{CTX-M-8}$ | F | TGATGAGACATCGCGTTAAG | 873 | [43] |
| | R | TAACCGTCGGTGACGATTTT | | |
| *bla*$_{CTX-M-9}$ | F | GTGACAAAGAGAGTGCAACGG | 857 | [43] |
| | R | ATGATTCTCGCCGCTGAAGCC | | |
| *bla*$_{CTX-M-10}$ | F | CCGCGCTACACTTTGTGGC | 944 | [43] |
| | R | TTACAAACCGTTGGTGACG | | |

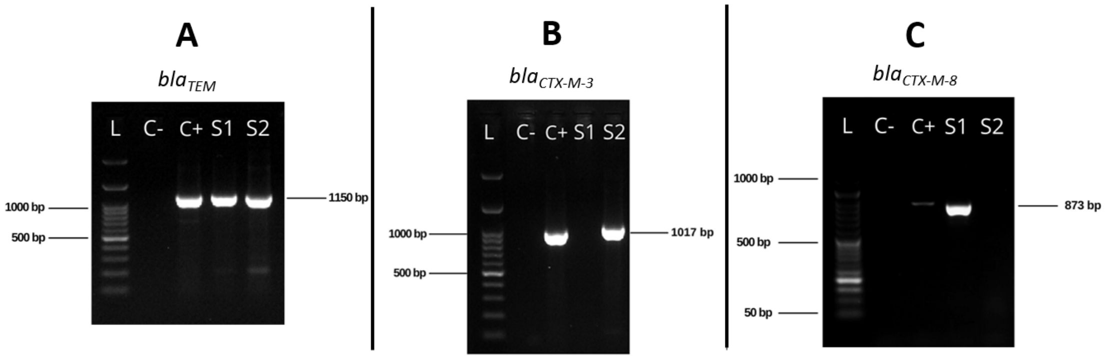


Figure S1: PCR amplification of β-lactamase genes harboring $bla_{TEM}$, $bla_{CTX\text{-}M\text{-}8}$ and $bla_{CTX\text{-}M\text{-}3}$ genes in Shigella spp. (S1) isolate from a dog faecal sample and E. coli (S2) isolate from a water sample. L, 100 bp DNA ladder, C-, negative control, C+, positive control. A) blaTEM (1150 bp), B) $bla_{CTX\text{-}M\text{-}3}$ (1017 bp), C) $bla_{CTX\text{-}M\text{-}8}$ (800 bp) amplification products.
